# The Vaginal Microbiota Composition and Genital Infections during and after Pregnancy among Women in Pemba Island, Tanzania

**DOI:** 10.3390/microorganisms10030509

**Published:** 2022-02-25

**Authors:** Naomi C. A. Juliana, Saikat Deb, Mohamed H. Juma, Linda Poort, Andries E. Budding, Abdalla Mbarouk, Said M. Ali, Sander Ouburg, Servaas A. Morré, Sunil Sazawal, Elena Ambrosino

**Affiliations:** 1 Institute for Public Health Genomics (IPHG), Department of Genetics and Cell Biology, Research School GROW (School for Oncology & Reproduction), Faculty of Health, Medicine & Life Sciences, University of Maastricht, 6200 Maastricht, The Netherlands; n.juliana@maastrichtuniversity.nl (N.C.A.J.); samorretravel@yahoo.co.uk (S.A.M.); 2 Public Health Laboratory—Ivo de Carneri, Chake Chake 74201, Pemba Island, Tanzania; saikatdeb@gmail.com (S.D.); mohduvumby@gmail.com (M.H.J.); omarabdallambaruok@gmail.com (A.M.); said@phlidc.org (S.M.A.); 3 Centre for Public Health Kinetics, New Delhi 110024, India; ssazawal@jhu.edu; 4 inBiome, 1098 Amsterdam, The Netherlands; linda.poort@inbiome.com (L.P.); dries.budding@inbiome.com (A.E.B.); 5 Laboratory of Immunogenetics, Department of Medical Microbiology and Infection Control, Amsterdam UMC, Location AMC, 1105 Amsterdam, The Netherlands; s.ouburg@microbenlab.com

**Keywords:** vaginal microbiota, genital pathogens, pathobionts

## Abstract

We investigated the vaginal microbiota (VMB) composition, prevalence of genital pathogens and their association among pregnant and post-delivery women in Pemba Island, Tanzania. Vaginal swabs were collected from 90 women, at two time points during pregnancy (<20 weeks of gestational age [GA] and ≥20 weeks GA) and once after delivery, when possible. IS-pro assay was used for VMB characterization. *Chlamydia trachomatis* (CT), *Neisseria gonorrhea* (NG), *Trichomonas vaginalis* (TV), *Mycoplasma genitalium* (MG) and human papillomavirus (HPV) were detected by qPCRs. VMB were mostly *Lactobacillus* dominant during pregnancy and non-*Lactobacillus* dominant post-delivery. A significant decrease in VMB richness was observed during pregnancy among paired and unpaired samples. Shannon diversity was significantly lower during pregnancy than post-delivery among unpaired samples. *Klebsiella* species and *Streptococcus anginosus* were the most commonly identified pathobionts at all timepoints. A high abundance of pathobionts was mostly seen in women with non-*Lactobacillus* dominant VMB. At ≥20 weeks GA timepoint during pregnancy, 63.0% of the women carrying one or more genital pathogen (either HPV, CT, TV, or MG) had *L. iners* dominant VMB. NG was not detected pre-delivery. This study contributes evidence on VMB composition, its changes during pregnancy and post-delivery, and their association with pathobionts and genital pathogens.

## 1. Introduction

The vaginal microbiota (VMB) consists of commensal microorganisms that exist in a mutually beneficial relationship with the host environment [1,2]. The human VMB are mostly dominated by protective lactic acid-producing *Lactobacillus* species in the majority of Caucasian women. These bacteria create an acidic environment with anti-microbial properties which hinders the growth and colonization of pathogenic microbial species [3,4,5]. Common *Lactobacillus* species in the vagina include *Lactobacillus* (*L.*) *iners*, *L. crispatus*, *L. gasseri*, *or L. jensenii* [2,6]. Jesper et al., also showed that next to the common *Lactobacillus* species, *L. vaginalis* plays a vital role in the VMB in African women [7]. However, when *Lactobacillus* species are in low abundance, women can carry higher levels of facultative anaerobic bacteria species, such as *Atopobium, Gardnerella, Ureaplasma, Bacteroides*, and *Prevotella* [8]. Furthermore, if the vaginal microbial community is dominated by non-lactic acid-producing species, it is less stable and tends to shift over time [9]. The polymicrobial anaerobic overgrowth disrupts the ecological balance of the VMB and is linked to vaginal anaerobic dysbiotic conditions like bacterial vaginosis (BV) [10,11,12,13]. BV is a common vaginal disorder in African women or women with a sub-Saharan African ethnic background [10,11] and has been associated with adverse outcomes such as miscarriage, premature rupture of membranes, preterm birth, and low birth weight [14,15,16]. The presence of pathobionts in the VMB, defined as potentially pathological organisms that generally live in a non-harming symbiosis, such as *Streptococcus agalactiae* (Group B streptococcus or GBS), *Staphylococcus aureus*, and species in the *Enterobacteriaceae* family, has also been associated with pelvic inflammatory disease or maternal and neonatal infections [17,18,19,20].

In individual women, the VMB composition differs between the non-pregnant and pregnant state. Indeed, the increased oestrogen and progesterone levels during pregnancy lead to physiological changes that also affect VMB composition [21]. Compared to the non-pregnant state, the VMB remain relatively stable during pregnancy, with an overall decrease in richness (number of species), abundance and evenness (relative abundance) of aerobic commensal bacteria and an increase in the abundance of *Lactobacillus* species from first to third trimester [7,8,22,23,24]. VMB changes in pregnancy mostly occur as transitions between species within the *Lactobacillus* genus, with rarely a shift to a polymicrobial state [22,25,26,27]. However, towards the end of the pregnancy and, especially, after delivery, following a decrease in oestrogen, a switch to a non-*Lactobacillus* dominant and more diverse VMB community is common [8,22]. This switch can persist up to one year postpartum [8,22]. *Lactobacillus* species-poor VMB and an increase in richness and diversity of VMB between 2nd and 3rd trimester have been associated with adverse pregnancy outcomes such as preterm birth, low birth weight, and miscarriage [28,29,30,31,32,33,34]. However, it is important to note that, although low abundances of pathobionts occur more often with lactobacilli than with BV-associated anaerobes, also women with *Lactobacillus* dominant VMB might have risk of developing adverse outcomes [35,36,37]. Thus, it might be that in certain individuals low abundance microorganisms might influence pregnancy progression more than dominant VMB species. To date, the role of VMB in preterm birth or other adverse pregnancy outcomes is controversial and still under investigation, especially since it seems to vary based on the ethnic background of the population studied. The composition of the VMB of women with African and non-African ancestry differs during pregnancy probably because of host genomics, immunological factors, microbial physiology and environmental influences [26,38,39,40]. Studies conducted on women with African ancestry observed that their VMB is less dominated by *Lactobacillus* species and more by BV-associated bacteria [6,26,30,33,41,42,43,44]. Several studies have investigated the VMB composition, irrespective of pregnancy status, across African populations, such as in Kenyan, Rwandan, South African, and Tanzanian women [45,46,47]. During pregnancy, it seems that *Lactobacillus*-dominant VMB (mostly *L. iners* and *L. crispatus*) are the most prevalent in women living in sub-Saharan Africa, followed by a more diverse VMB composition [48]. However, in the study conducted in mainland Tanzania, the VMB bacteria were only characterized on genus level and not species level [49].

Due to the possible impact that certain VMB and genital microorganisms have on health (reproductive and pregnancy outcomes) it is important to investigate them also in populations where the burden of disease is highest. In sub-Saharan Africa there is not only a high burden of vaginal dysbiotic conditions (such as BV), but also genital infections (such as sexually transmitted infections) and adverse pregnancy outcomes [50,51,52,53,54]. The interaction between VMB, genital pathogens and pathobionts during pregnancy is complex and remains mostly unclear [20,55]. Moreover, data about the VMB composition and presence of pathobionts in the sub-Saharan African population, especially Tanzania, are still limited. In 2014, a biobanking effort was initiated in Pemba Island, Tanzania with the support of the Bill and Melinda Gates Foundation [56]. Within this previously established (AMANHI) biobanking effort, adverse pregnancy outcomes and vaginal samples were collected during pregnancy and after parturition. The aim of this study was to characterize, in a small sub-set of samples, the VMB composition and its changes, including the presence of pathobionts and genital infections, across two timepoints during pregnancy and once after delivery using the previously collected biobank data and samples. It was hypothesized that the most prevalent VMB composition would be *Lactobacillus*-dominant VMB, with high frequency of a diverse VMB composition and presence of pathobionts. Insights from this first attempt to longitudinally characterize the VMB composition in Pemban women will contribute evidence on the role of microorganism in maternal and neonatal health among sub-Saharan African women.

## 2. Materials and Methods

### 2.1. Samples and Study Design

Vaginal sample collection was performed in the context of a biobanking effort established with the support of the Bill and Melinda Gates Foundation and initiated in 2014 as part of the Alliance for Maternal and Newborn Health Improvement (AMANHI) [56]. All women included in this study gave their prior consent, and data collection was conducted as per protocol [56]. Vaginal swabs collection was performed at 2 timepoints during pregnancy and once after delivery under health care staff supervision in health care facilities in Pemba Island [56,57]. Early pregnancy dating ultrasound determined the gestational age (GA) [52]. The first timepoint of sample collection during pregnancy was between 8–19 weeks and 6 days GA (<20 weeks), the other timepoint during pregnancy was between 20–40 GA weeks (≥20 weeks GA), and samples were collected between 42–60 days post-delivery. The cut-off of 20 weeks GA also refers to a clinically relevant time during pregnancy, after which spontaneous pregnancy loss, referred to as miscarriage, is usually less frequent. The timing of collection was not standardized; women were not sampled at all timepoints—this largely depended on practical limitations in rural clinics. Baseline sociodemographic and previous health care information was collected by health care staff at first antenatal contact. In addition, at each later timepoint further health information was collected by health care staff following the previous published protocol [56]. Swabs were stored in 1 mL eNAT buffer (Copan Italia, Brescia, Italy) at −20 °C at the Public Health Laboratory—Ivo de Carneri in Pemba Island. The collection tubes with eNAT buffer were later transported in dry-ice to Amsterdam Medical Centre in the Netherlands, where they were stored at −20 °C until further processing [58]. In this study, vaginal swabs collected at one or more of the 3 collection timepoints up until January 2019 were analysed. The Zanzibar Medical Research and Ethics Committee (ZAMREC) approved this study (protocol ZAMREC/0002/OCTOBER/013 amended 04/02/18).

### 2.2. Dna Extraction and Vaginal Microbiota Analysis

DNA from the vaginal swabs was extracted with the Chemagen (Perkin-Elmer, Baesweiler, Germany) automated DNA extraction machine according to the buccal swab extraction kit manufacturer’s instructions, as previously described elsewhere [59]. Elution volume was 200 μL. The IS-pro Microbiota assay (inBiome, Amsterdam, The Netherlands) was used for the VMB analysis [59]. The assay is based on length polymorphisms of the 16S–23S interspace (IS) region combined with sequence polymorphisms of the 16S rDNA. The IS-pro assay consists of two multiplex PCRs and was performed according to the manufacturer’s protocol as previously described [59,60,61]. In short, the first PCR used two different fluorescent-labelled primers, one for the phyla *Bacteroidetes*, and *Fusobacteria, Actinobacteria, Firmicutes,* and *Verrucomicrobia* (FAFV), while the second PCR included primers for the phylum *Proteobacteria*. An internal amplification control (IAC) was used for quality control of the process and downstream software analyses. After DNA amplification with the GeneAmp PCR system 9700 (Applied Biosystems, Foster City, CA, USA), 5 μL of PCR product was mixed with 20 μL formamide and 0.5 μL MapMaker 1500 ROX-labelled size maker (BioVentures, Murfreesboro, TN, USA). The ABI Prism 3500 Genetic Analyzer (Thermo-Fisher, Waltham, MA, USA) was used for the DNA fragment analysis via high-resolution capillary electrophoresis. Species were assigned to resulting amplicon length and colour using a reference database compiled of IS-pro fragments obtained from in silico and in vitro IS-pro PCRs of known vagina associated bacterial species (species calling). The IAC tested positive for all samples. TIBCO Spotfire 7.6 (TIBCO Spotfire Inc., Palo Alto, CA, USA) software was used to visualize the IS-pro colour labelled nucleotide peaks, species and to cluster the sample profiles according to the unweighted pair-group with the arithmetic mean (UPGMA) method. To cluster the microbiome profiles based on similarity column correlation, the process was performed with the UPGMA on a similarity matrix based on cosine similarity of bacterial profiles. Row hierarchical clustering was done by UPGMA on a distance matrix based on Euclidean distance. It identifies and orders the most frequent IS-fragments or bacterial taxa related to the microbiome profiles. For the purpose of this study, six bacterial genera (*Streptococcus, Staphylococcus*, *Enterococcus, Escherichia/Shigella*, *Haemophilus*, and *Campylobacter*) are considered pathobionts, as suggested by Wijgert et al. [20]. Pathobionts with a relative abundance higher than 20% are considered to be of substantial presence in the VMB profile [20]. After the hierarchical clustering of vaginal microbiome profiles, five microbial communities in line with the previously defined Community State Types (CST) were identified [6]. Alpha diversity indices of the VMB were measured by calculating the richness (number of species) and the Shannon diversity (the richness and relative abundance of bacterial species) index of each sample on the Spotfire software [62].

### 2.3. Genital Pathogens Analysis

Presence of *C. trachomatis, n. gonorrhoeae* and *T. vaginalis* was detected by their respective CE-IVD certified Presto and Real-Time quantitative polymerase chain reaction (qPCR) with ABI Taqman 7500 (Applied Biosystems, Foster City, CA, USA) according to the manufacturer’s instructions, and as previously described [63,64]. For *M. genitalium* detection, a *M. genitalium* assay targeting the mg219 gene was used on the LightCycler 480 II PCR machine (Roche Diagnostics, Basel, Switzerland) [58,65]. The presence of high-risk human papillomavirus (hrHPV), which might be associated with different degrees of pathophysiology and local inflammation, was detected. To do that, AmpFire^®^ HPV assay was used according to the user manual instruction, to simultaneously identify either HPV 16 genotype, HPV 18 genotype or fifteen other hrHPV genotypes (31, 33, 35, 39, 45, 51, 52, 53, 56, 58, 59, 66 and 68, not individually identified and further referred in this manuscript as HPV others) [66].

### 2.4. Statistical Analysis

Data were analysed using IBM SPSS statistical software version 26 (SPSS Inc., Chicago, IL, USA). Fisher’s exact test was performed to compare dichotomous data in order to test whether the presence of genital infections associated with CST III compared to other CSTs (I, II, IV, V) [67,68]. To test whether alpha diversity indices were significantly different across time points, the Wilcoxon signed rank test (for matched samples) or Mann-Whitney U-test (for unmatched samples) was used. Whitney U-test (for unmatched samples) was used to calculate significant differences in relative abundances for pathobionts across timepoints. A two-tailed *p*-value of < 0.05 was considered statistically significant.

## 3. Results

In total, 170 vaginal samples from 90 Afro Shirazi women were included for this analysis. Forty-four samples were collected between 8–20 weeks gestational age (GA), eighty-two samples between 20–40 weeks GA, and forty-four samples between 42–60 days post-delivery (Supplementary Figure S1). Seventy-eight women underwent sample collection at two timepoints (either both during pregnancy or once during pregnancy and the other post-delivery) and two women at three timepoints. Questionnaires have been filled by most participants at baseline and other collection timepoints (Table 1, Table 2 and Table 3).

### 3.1. Sociodemographic Characteristics and Birth Data

The sociodemographic and health-related questions were filled out by more than 85% of the 90 participants (Table 1). The mean maternal age of the participants was 29.9 ± 6.6 years, mean gravidity 5.2 ± 2.6, and mean parity of 4.0 ± 2.4. The majority of the women were multiparous (93.2%). Almost half of the participants had a healthy weight (44.4%). None of the participants smoked at the time of enrolment, and 96.6% were not on any dietary restrictions (*n* = 88). At enrolment, the most common obstetric history complication reported was miscarriage/abortion (29.3%), followed by stillbirth (12.2%), preterm birth (3.8%) and premature rupture of membranes (PROM) (2.5%). Two of the 88 women (2.3%) reported they had malaria at the time of enrolment. Other medical problems or infections (diabetes, thyroid disease infections with human immunodeficiency virus (HIV), tuberculosis, hepatitis B or C, and urinary tract infection) were not reported.

The information collected might have been different per timepoint, depending on the number of women that responded to the questionnaire item (Table 1, Table 2 and Table 3). At the time of ≥ 20 weeks GA sampling during pregnancy, 34 (43%) women self-reported the use of multivitamins or iron and other minerals, and four (5.1%) women self-reported that they had taken antibiotics during their pregnancy. Some participants reported gums or teeth complaints (2.5% and 6.3%, respectively), but did not report any other health condition (haemorrhage, bleeding, diabetes, thyroid disease, HIV, malaria, tuberculosis, jaundice, hepatitis B or C, and urinary tract symptoms) (*n* = 79). At the time of sampling post-delivery, 78 women filled in the summary and one (1.3%) woman self-reported she had diabetes during her pregnancy. No other medical conditions or infections that might have occurred during the current pregnancy were reported (thyroid disease, cancer, cardiac problems, epilepsy, mental illness, hypertension HIV, malaria, tuberculosis, jaundice, hepatitis B or C and urinary tract symptoms). Five women (6.4%) reported that they had received antibiotics and 26 (33.3%) women reported they had received other types of medicines (not specified). The gestational time at vaginal swab collection, pregnancy data, pregnancy outcomes and neonatal outcomes are described in Table 2. The majority of the women (89%) received assistance at birth either by a doctor, nurse or midwife (*n* = 90). One participant experienced a stillbirth, four women a miscarriage, six women had a preterm delivery (<37 GA weeks); the rest had an uncomplicated birth (Table 3). Two women reported that their neonates were ill within the first 48 h of life; however, the type of illness was not further specified. The pregnancy outcomes of the women that had vaginal samples collected post-delivery are described in Table 3.

### 3.2. Species in the Vaginal Microbiota

The fluorescent colour and the length of the IS region in units of nucleotides per sample were identified in three groups at the phyla level, namely *Bacteroidetes*, FAFV, and *Proteobacteria* (Supplementary Figures S2–S4). In the included samples from 90 women, 554 different bacterial species were detected. Across the combined vaginal samples, *Lactobacillus* species (*L. crispatus*, *L. iners*, and *L. jensenii*) or *Klebsiella* species were the most identified species of the VMB during pregnancy (Supplementary Figure S4). In contrast, in post-delivery samples, the most identified species were *L. iners*, *Gardnerella vaginalis*, *L. crispatus*, and *Klebsiella* species (Supplementary Figure S5).

### 3.3. Shannon Index and Diversity

**Figure 1:**
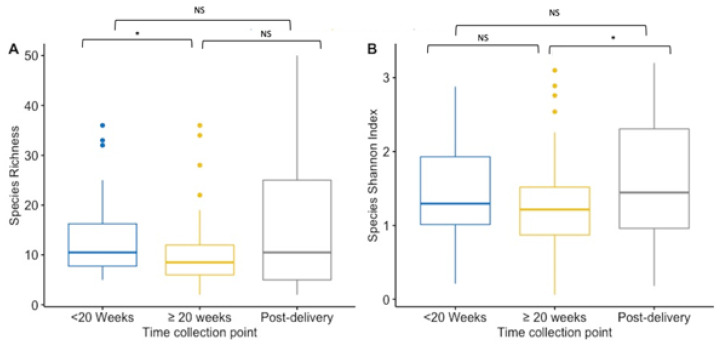
Boxplot for the richness (**A**) and Shannon diversity index (**B**) at the species level for each collection point. Results of the <20 weeks GA pregnancy collection are in blue (*n* = 44), ≥20 weeks GA pregnancy collection in yellow (*n* = 82), and post-delivery in grey (*n* = 44). (**A**) Species richness is lower at ≥20 weeks GA pregnancy collection compared to the pregnancy collection at <20 weeks GA (*p* = 0.02). Between the other timepoints there was no significance difference. (**B**) The Shannon diversity index is higher at post-delivery compared to the index at ≥20 weeks GA pregnancy collection (*p* = 0.03), but not compared to the diversity index at <20 weeks GA pregnancy collection. During the pregnancy, the index did not differ significantly. NS = non significant. * *p*-value < 0.05.

Alpha-diversity indices (richness and Shannon diversity index) of the VMB community were compared between paired and unpaired vaginal samples collected at first and ≥20 weeks GA pregnancy timepoint, and post-delivery for species and IS-fragment count. Data on IS-fragment count are shown in the Supplementary Figures S7 and S9. When performing unpaired analysis, species richness was significantly lower during pregnancy (from mean 10 species to mean 13 species) (*p* = 0.02) (Figure 1A). While the mean richness (15.39 species) was the highest post-delivery, there were no significant differences in the mean richness in the unpaired vaginal samples collected at enrolment or at the ≥20 weeks GA pregnancy sampling collection (Figure 1A). During pregnancy, the Shannon index did not differ significantly between the two collection points in the unpaired vaginal samples (mean Shannon diversity index was 1.42 at <20 weeks GA timepoint compared to 1.23 at ≥20 weeks GA timepoint) (Figure 1B). However, the mean Shannon diversity index was significantly higher in post-delivery collected unpaired vaginal samples (mean Shannon diversity index = 1.62) compared to the ≥20 weeks GA pregnancy collection point (*p* = 0.03), but not significantly higher compared to the <20 weeks GA timepoint (Figure 1B).

**Figure 2:**
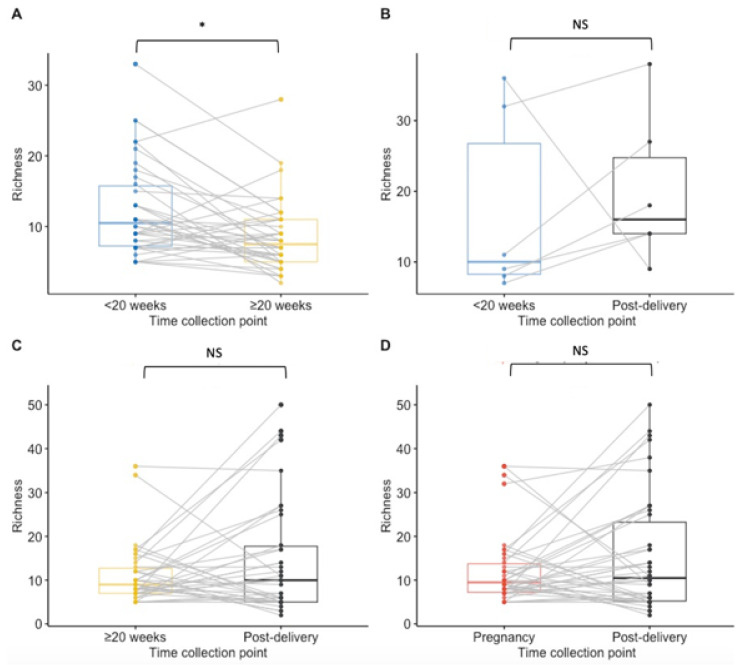
Boxplots for species richness at each collection point for paired samples. Results of <20 weeks GA pregnancy collection are in blue, ≥20 weeks GA pregnancy collection in yellow, post-delivery in black and overall during pregnancy in red. (**A**) The richness was significantly lower at the ≥20 weeks GA pregnancy collection point than at <20 weeks GA pregnancy collection (*p* = 0.02) in matched samples from 38 women. (**B**) There was no significant difference in the richness between <20 weeks GA pregnancy collection and post-delivery matched samples from 6 women. (**C**) No significant difference was calculated in the richness between ≥20 weeks GA pregnancy collection and post-delivery matched samples from 38 women. (**D**) For 42 women that had samples collected at least once during pregnancy and post-delivery, no significant difference in the species richness was calculated. NS = non significant. * *p*-value < 0.05.

**Figure 3:**
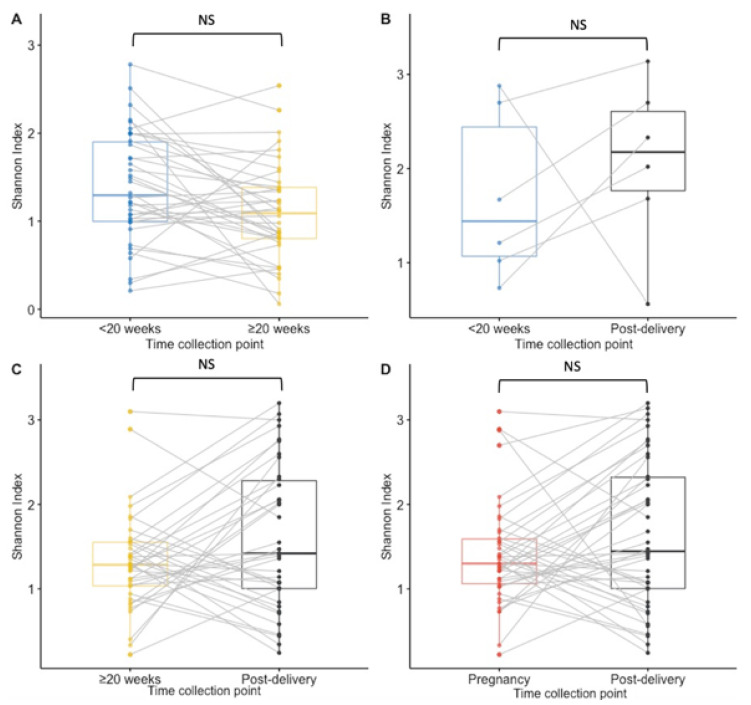
Boxplots for the Shannon diversity index at species level for paired samples. Results of <20 weeks GA pregnancy collection are in blue, ≥20 weeks GA pregnancy collection in yellow, post-delivery in black and pregnancy in red. (**A**) The Shannon diversity index was not significantly higher at the ≥20 weeks GA pregnancy collection point than at the <20 weeks GA pregnancy collection in matched samples from 38 women. (**B**) There was no significant difference in the Shannon diversity index between the <20 weeks GA pregnancy collection and post-delivery matched samples from 6 women. (**C**) No significant difference was calculated in the Shannon diversity between the ≥20 weeks GA pregnancy collection and post-delivery matched samples from 38 women. (**D**) For 42 women who had samples collected at least once during pregnancy and post-delivery, no significant difference in the Shannon diversity index was calculated. NS = non significant.

Paired analysis of samples from 38 women tested at both timepoints during pregnancy showed a significant decrease in richness during pregnancy (median < 20 weeks GA timepoint = 10.5 species; median ≥ 20 weeks GA collection pregnancy point = 7.5, *p* = 0.02) and no statistically significant difference between other time points (Figure 2A–D). The Shannon index findings across timepoints using paired analysis are similar to the Richness findings, with only a significant decrease in diversity observed during pregnancy (Figure 3A,B,D). Unlike in the unpaired analysis, for the paired analysis the Shannon diversity index did not significantly increase when analysing data of a subset of 38 other women that were tested both at the ≥20 weeks GA timepoint during pregnancy and post-delivery (*p*-value = 0.068) (Figure 3C).

### 3.4. Community State Types

**Figure 4:**
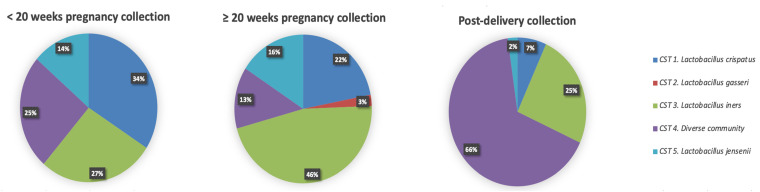
Frequency of five community state type (CST) identified each collection timepoint (*n* = 44 at <20 weeks GA and post-delivery timepoints; *n* = 82 at the ≥20 weeks GA timepoint). In the pie charts the CST cluster is given in colour: CST I, blue; CST II, green; CST III, red; CST IV, yellow; CST V, purple.

**Figure 5:**
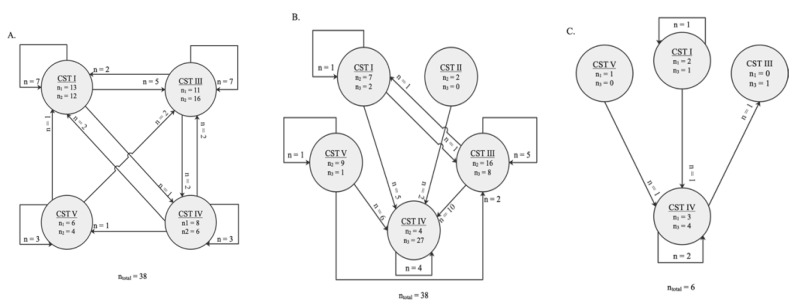
Schematic representation of the switch between community state types (CSTs) between two sampling timepoints. The numbers in the circles indicate how many vaginal samples were clustered in a certain CST by the time collection (during pregnancy). Arrows represent the direction of the switch. The numbers by the arrows represent the number of vaginal microbiota tested belonging to the same women who switched to a given CST at a later timepoint. (**A**) Thirty-eight women were clustered with a specific CST at timepoint <20 weeks GA and ≥20 weeks GA during pregnancy, of which 18 CST changed type during pregnancy. (**B**) Among the paired vaginal swabs tested at ≥20 weeks GA and post-delivery, 27 of the 38 CST changed. (**C**) The CST of three of the vaginal swabs from the same six women tested at <20 weeks GA and post-delivery changed.

Hierarchical clustering of VMB profiles from samples collected during pregnancy and post-delivery yielded five different CSTs as previously described by Ravel et al. [6] (Figure 4). The VMB profiles of the vaginal samples collected at the <20 weeks GA timepoint during pregnancy (*n* = 44), were dominated by *Lactobacillus* species (33/44; 75%) belonging to three different CSTs: CST I (*L. crispatus*, 15/44; 34%), CST III (*L. iners*, 12/44; 27%), and CST V (*L. jensenii*, 6/44; 14%), respectively (Figure 4). The rest of the women (11/44; 25%) had a diverse VMB clustered as CST IV. Compared to samples collected at the < 20 weeks GA timepoint, the VMB profiles of the vaginal samples collected at the ≥ 20 weeks GA pregnancy timepoint (*n* = 82) were less diverse (CST IV; 11/82; 13% compared to 25%), and more vaginal samples (71/82; 87% compared to 75%) had a *Lactobacillus* dominant profile belonging to: CST I (18/82;22%), CST II (*L. gasseri*, 2/82; 2%), CST III (38/82; 46%), and CST V (*L. jensenii*, 13/82; 16%) (Figure 5), respectively. Due to the limitations of small sample size, the mentioned comparison between the < 20 weeks GA and the ≥ 20 weeks GA timepoint is purely descriptive and has not been statistically tested. Most of the vaginal samples collected post-delivery had a diverse VMB profile (CST IV; 29/44; 66%) followed by a *L. iners* dominated VMB (CST III; 11/44; 25%) (Figure 4). The remaining vaginal samples were clustered in CST I (3/44; 7%) and CST V (1/44; 2%).

Among the six women who had preterm deliveries, during pregnancy two of them had CST I (33%), two CST IV (33%), one CST V (17%) and another had CST III (17%). The two women carrying twins both had vaginal samples belonging to CST I at the <20 weeks GA timepoint during pregnancy. One of the women with multiple gestation also had CST I at the ≥20 weeks GA timepoint; after parturition the VMB profile switched to CST IV. The other participant switched from CST I to CST III at the ≥20 weeks GA timepoint; unfortunately there was no vaginal sample available post-delivery. The only woman who reported a stillbirth delivery had a vaginal sample belonging to CST III at the second timepoint. The vaginal profile at the first timepoint and post-delivery could not have been analysed due to the unavailability of these vaginal swabs.

For the four women who had a miscarriage, two vaginal samples at the <20 weeks GA pregnancy collection belonged to CST IV, one to CST III and another to CST I. Vaginal profiles from three women were also analysed at the post-delivery timepoint; one woman had CST I, another women CST IV, and another women CST III.

Furthermore, four women reported antibiotics usage at the ≥20 weeks GA pregnancy collection timepoint. At that timepoint, their vaginal samples belonged to CST III (*n* = 2), CST I (*n* = 1), CST IV (*n* = 1).

Overall, in the tested samples, changes of vaginal profiles occurred during pregnancy and after delivery (Figure 5). During pregnancy, the VMB communities shifted from one CST to another in 18 of the 38 (47%) vaginal samples that were analysed between the <20 weeks GA and the ≥20 weeks GA timepoint during pregnancy (Figure 5A). Between the two timepoints during pregnancy, a switch to CST III accounted for 9 (50%) of these changes. The CST shifted in 27 of the 38 women (71%) whose vaginal profiles were analysed at the ≥20 weeks GA timepoint during pregnancy and post-delivery (Figure 5B). Moreover, the CST shifted in 3 out of 6 (50%) vaginal profiles that were analysed at the <20 weeks GA timepoint in pregnancy and post-delivery (Figure 5C). From pregnancy to post-pregnancy timepoints, a switch to CST IV accounted for 23 (85%) of these changes. While CST I and CST III were the most interconnected states across longitudinal timepoints, with bidirectional transitions with each other and other CSTs (mostly CST IV) (Figure 5A–C).

### 3.5. Vaginal Pathobionts and Genital Pathogens

**Figure 6:**
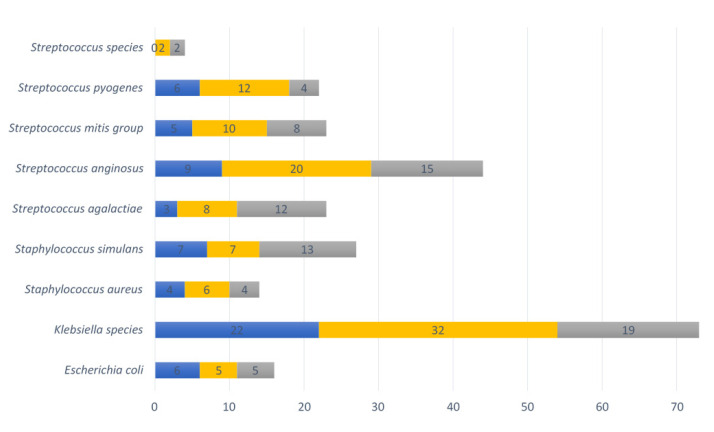
Nine pathobionts observed in vaginal samples per collection point. Numbers of vaginal samples detected with pathobionts at <20 weeks GA pregnancy collection (*n* = 44), ≥20 weeks GA pregnancy collection (*n* = 82), and post-delivery (*n* = 44). Blue refers to the first timepoint during pregnancy, yellow to the second timepoint during pregnancy and grey to the post-delivery timepoint.

The following bacterial species of the VMB with pathogenic potential were identified: *Escherichia coli*, *Klebsiella* species, *Staphylococcus aureus*, *Staphylococcus simulans*, *Streptococcus agalactiae*, *Streptococcus anginosus*, *Streptococcus mitis* group, *Streptococcus pyogenes*, and *Streptococcus* species (other than already mentioned). In total, 73% (32/44) of vaginal swabs contained at least one such pathobiont at the <20 weeks GA timepoint during pregnancy, 68% (58/82) at ≥20 weeks GA, and 86% (38/44) post-delivery. The most common pathobiont at all three timepoints was the Klebsiella species, followed by *Streptococcus anginosus*, *Streptococcus pyogenes*, the *Streptococcus mitis* group, *Staphylococcus simulans* and *Streptococcus agalactiae* (Figure 6).

Three samples containing pathobionts at the <20 weeks GA timepoint (3/58) also had a substantial presence (>20% relative abundance, range between 22 and 35%) of pathobionts (two samples with *Klebsiella* species, and one sample with *Streptococcus mitis* group and *Streptococcus anginosus*). Among these samples, the sample with a relative abundance above 30% with the pathobiont *Klebsiella* species belonged to CST III, while the other two samples belonged to CST IV. At the ≥20 weeks GA time point, seven vaginal samples (7/38) had a substantial (relative abundance range: 20–44%) presence of pathobionts at the ≥20 weeks GA timepoint (*Klebsiella* species [*n* = 2], *Streptococcus agalactiae* [*n* = 1], *Streptococcus anginosus* [*n* = 3] and *Staphylococcus simulans* [*n* = 1]). Among those samples with high pathobionts load, two had a VMB belonging to CST III, while the others belonged to CST IV (*n* = 4) or CST V (*n* = 1).

Nine vaginal samples (9/38) had a substantial presence of pathobionts (relative abundance 24–89%) at the post-delivery collection point. Two different vaginal samples had a combination of a high relative abundance of *Staphylococcus simulans* (>30%) and *Streptococcus anginosus* (>44%). The rest of the samples had a high relative abundance of *Klebsiella* species (42%), *Streptococcus anginosus* (26% and 32%) and *Streptococcus agalactiae* (24%, 69%, 76%, and 89%). All nine post-delivery samples with a substantial presence of pathobionts belonged to CST IV. The overall relative abundance of pathobionts was significantly higher in the samples collected post-delivery (mean 10.6%) than in samples collected during pregnancy (<20 weeks GA pregnancy collection mean 6.0%; *p*-value = 0.04, ≥20 weeks GA pregnancy collection mean 4.9%; *p*-value = 0.01).

The known genital pathogens *C. trachomatis*, *T. vaginalis*, *M. genitalium*, *N. gonorrhoeae* and hrHPV other than genotypes 16 or 18 were also detected in the samples (Table 4). Nine samples (20.5%) were positive for at least one genital pathogen at the <20 weeks GA timepoint (Table 4). *C. trachomatis* was detected in four vaginal samples, each belonging to CST I, CST III, CST IV, and CST V, respectively. The two vaginal samples that were positive for *T. vaginalis* at the <20 weeks GA timepoint belonged to CST I and CST III, while three others were positive for hrHPV genotypes and belonged to CST I, CST III or CST V, respectively (Table 4). The presence of urogenital pathogens at the <20 weeks GA timepoint during pregnancy did not cluster significantly more within CST III (Table 4).

At the ≥20 weeks GA timepoint during pregnancy, 18 (22.8%) vaginal samples tested positive for one or more genital pathogens (Table 4), most of them belonged to CST III (*n* = 12), followed by CST I (*n* = 2), CST V (*n* = 2), CST II (*n* = 1), and CST IV (*n* = 1). Most of the samples with a genital pathogen at the ≥ 20 weeks GA timepoint belonged to CST III (12/38); this difference was statistically significant (*p* = 0.01) when comparing all combined pathogens across CSTs (6/38).

Of the 44 post-delivery samples, five (11.4%) were positive for *C. trachomatis* (*n* = 1), three (6.8%) for *T. vaginalis*, one (2.3%) for *N. gonorrhoeae*, or four (9.1%) for hrHPV genotypes. The sample positives for a genital infection were clustered either in CST III along or CST IV (Table 4). The results of the persistence of genital infection during and after pregnancy in a larger cohort of this population is described elsewhere [57].

## 4. Discussion

Using vaginal samples and questionnaire data previously collected from a biobank in Pemba Island, this study analysed VMB and genital infections among 90 local Afro-Shirazi women during pregnancy and post-delivery. This study strived to comprehensively characterize the vaginal microbial environment, by identifying the bacterial species present (including pathobionts), by using this information to cluster the VMB into CSTs, and by identifying the presence of selected genital pathogens. During pregnancy, the VMB communities were mostly *Lactobacillus*-dominated with most VMB profiles belonging to CST I, II, III, and V (65% of the vaginal samples at the <20 weeks GA timepoint and 81% of the vaginal samples at ≥20 weeks GA [8]). These results are in concordance with previous (longitudinal) observations where a high relative abundance of *Lactobacillus* species was observed during pregnancy, and *L. crispatus* (CST I) and *L. iners* (CST III) were the most commonly identified species of the VMB community during pregnancy [23,25,69,70]. In particular, alike to previous studies among African and African-American pregnant women, this study also showed the presence of *L. jensenii* (CST V) or *L. gasseri* (CST II), though most often not as dominant *Lactobacillus* species [7,23,45]. These observations during pregnancy in this and other studies are in discordance with the studies of non-pregnant Tanzanian women and another cohort of sub-Saharan African women [7,23,45,47].

Across the two timepoints in pregnancy, Shannon diversity index, a measure of alpha diversity, of VMB was stable, and the richness was significantly lower (*p* = 0.02) than after pregnancy, as previously observed by other longitudinal studies [25]. Similar to a study in North American women from African ancestry, the prevalence of *Lactobacillus*-dominated VMB in this current study was lower at the <20 weeks GA timepoint than in later trimesters of pregnancy, and the switch in VMB in pregnancy was most commonly (50% [9/18] of cases) towards *L. iners* dominant VMB [26]. The high frequency of a *Lactobacillus* dominant VMB and the stability of the alpha diversity in pregnancy has been attributed to the high levels of oestrogens that indirectly promote glycogen production and support *Lactobacillus* species colonization, which in turn metabolize lactic acid and promote a healthy low vaginal pH [23]. This hypothesis might explain the predominant role of high oestrogens levels during pregnancy, independent of ethnicity, in promoting *Lactobacillus* abundance. In the non-pregnant state, on the other hand, when oestrogens’ role is not as important, the frequency of the non-*Lactobacillus* dominated CST IV has been reported to be higher in women from sub-Saharan Africa or with African ancestry [26,45,71,72].

After delivery, a 100- to 1000-fold drop of oestrogens and resulting disruption of the vaginal microbial environment is expected [8,43,73,74], including the oestrogen-driven *Lactobacillus* species dominance [43]. In agreement with these data, the study results showed that, in paired samples, most vaginal profiles shifted to CST IV post-delivery. In 74% of the vaginal samples collected post-delivery the VMB profile were more diverse, belonging to CST IV, and were less *Lactobacillus* dominant than VMB during pregnancy. This observation agrees with previous ones among British women of different ethnic backgrounds and among American women. Such changes have been reported to persist up to one year, independent of ethnicity [8,43]. Mean and median richness and Shannon diversity index were higher in the post-delivery samples compared to samples collected during pregnancy, though this difference was only statistically significant for the mean Shannon diversity index calculated for unpaired samples (*p* = 0.03). The median Shannon diversity index calculated for the paired samples collected at the ≥20 weeks GA pregnancy timepoint and post-delivery was close to statistical significance (*p* = 0.07). Nevertheless, these findings combined suggest that the VMB diversity and richness during pregnancy is lower than post-delivery in women from Pemba Island, Tanzania. These findings are also in concordance with previous observations in post-delivery VMB analysis from mainland Tanzanian, British and American women, in whom the authors also described a more diverse and richer VMB throughout pregnancy [8,43,49]. Also similar to other studies, an increase in BV-associated bacteria such as *Gardnerella vaginalis, Prevotella* species and *Anaerococcus* species, was also observed post-delivery [8,43,49]. The presence of *Prevotella* species and *Anaerococcus* species have been associated with a higher vaginal pH and BV [6,75]. In this present study, the presence of clinical BV could not be assessed, as this was not addressed in the questionnaire. However, in the questionnaires, none of the women reported any urinary tract infections symptoms, which often present as symptoms of the urogenital tract [58]. Thus, it is possible that most women in this cohort with a dysbiotic (CST IV) VMB were asymptomatic [11]. However further studies should investigate the exact prevalence of BV and BV-symptomatology in this Tanzanian population. Nonetheless, even though mostly asymptomatic, the role of certain BV-associated species during pregnancy is gaining increasing interest as BV-associated species have been associated with preterm birth in Caucasian cohorts [8,30,76]. However, a more extensive African-American study found no specific taxon, including *Gardnerella*, as a significant marker for preterm birth [23,30]. Further research is needed to investigate the role of BV-associated bacteria in sub-Saharan African cohorts, especially since the burden of preterm birth is high in this world region [77].

The presence of certain vaginal commensal microorganisms (pathobionts), such as *S. agalactiae, Staphylococcus aureus* and species in the *Enterobacteriaceae* family under certain circumstances can be also of clinical relevance in various maternal, pregnancy-related and neonatal health conditions [17,18,78,79]. In the samples tested in this study, pathobionts were more present post-delivery (86%) than during pregnancy (68% at the <20 weeks GA timepoint and 73% at the ≥20 weeks GA timepoint in pregnancy). Moreover, post-delivery, more women (20%) had a VMB containing a substantial presence of pathobionts (relative abundance more than 20%) compared to VMB from samples collected during pregnancy (6.8% at the <20 weeks GA and 8.5% at the ≥20 weeks GA timepoint during pregnancy). However, it is for debate whether the risk of pathogenic potential increases only when the VMB contains a substantial presence of a pathobiont (higher abundance) [20]. As previously observed in the non-pregnant cohort studies by Wijgert et al., in this study, pathobionts co-occur with both lactobacilli and BV-associated bacterial species during pregnancy [20]. However, post-delivery most pathobionts were detected in women with a dysbiotic vaginal profile (CST IV). The causal association between CST IV and pathobionts’ abundance, along with their role in pregnancy complications, such as preterm birth, should be further investigated.

*Klebsiella* species and *Streptococcus anginosus* were the most prevalent pathobionts during pregnancy and post-delivery, and in most cases, they were also the most prevalent pathobionts with a substantial presence in the VMB. The prevalence of *Klebsiella* species during pregnancy (2.3% at <20 weeks GA and 4.5% at the ≥20 weeks GA timepoint) were similar in this study compared to previous findings in pregnant Nigerian or Nepalese women where the point-prevalence was 3% or 5.6%, respectively [80,81]. *Streptococcus anginosus* is one of most identified pathobionts in patients with aerobic vaginitis, together with other pathobionts (including *Escherichia coli, Streptococcus* spp., *Staphylococcus aureus, Staphylococcus epidermidis*) [82]. The exact role of *Streptococcus anginosus* in the female genital tract and the influence of aerobic vaginitis in pregnancy is still unknown; however, there have been cases where fatal neonatal sepsis was reported to occur due to a specific *Streptococcus anginosus* biotype [79,83].

There are also indications that *K. pneumoniae* in particular is associated with neonatal deaths and premature pregnancy loss [84]. Thus, identifications of these pathogens (also in substantial presence) in this cohort indicate the need to further investigate in larger studies the clinical association between *Klebsiella* species and *Streptococcus anginosus* and adverse pregnancy outcomes.

Currently, in certain parts of the world, especially high-income countries, *Streptococcus agalactiae*, also known as GBS, is tested in pregnancy as part of antenatal care to minimize the risk of vertical infection to the foetus and new-born [85]. GBS infection can lead to preterm birth and can be fatal for neonates, causing severe cases of pneumonia, meningitis or sepsis [17,18,86]. The prevalence of GBS observed in this study (6.8% at <20 weeks GA, 10% at the ≥20 weeks GA timepoint in pregnancy, and 27% post-delivery) are in line with the pooled prevalence of a recent meta-analysis report on the prevalence of GBS in pregnant and post-delivery women in Tanzania (16.14%; 95% CI 2.9, 29.4) [87]. It should be noted that about 1 in 100 infants born from mothers carrying GBS develop invasive GBS infection disease in studies performed on other populations [88,89]. Thus, as evidence on the involvement of pathobionts in maternal and new-born health, and their possible vertical transmission, it is essential to further investigate the association between abundance of GBS, and other pathobionts, and perinatal health. It is even more so, now that some of these pathobionts strains, like *Klebsiella* and *E. coli*, are becoming increasingly resistance to a broad range of antibiotics [81,89].

In addition to pathobionts, this study also investigated the presence of the pathogens *C. trachomatis*, hrHPV, *M. genitalium*, and *T. vaginalis* in this cohort. In total, 21%, 23%, and 11% of vaginal samples tested positive for a genital pathogen at the <20 weeks GA and ≥20 weeks GA timepoints during pregnancy and post-delivery, respectively. It has been proposed that a VMB characterized by low diversity of species has a protective role against ascending infections of the genital tract and adverse pregnancy outcomes, and that infections in early pregnancy may hinder the transition of VMB to a beneficial state associated to a lower risk of pregnancy complication, i.e., CST I [23,67,90]. *T. vaginalis* has been associated with reduced *Lactobacillus* species in the VMB, but not BV-related species [87,88]. In contrast, *L. iners* has been associated with susceptibility to genital infections (HIV and other sexually transmitted infections) [67,68,91,92]. In this study, The VMB samples of most women carrying a genital pathogen belonged to CST III (*L. iners* dominant) at the ≥ 20 weeks GA pregnancy timepoint. However, at the other timepoints, the presence of none of the genital pathogens clustered with VMB belonging this specific CST. To this date, the role of *L. iners* in the VMB equilibrium is not fully understood, as it has been associated with both eubiotic and dysbiotic VMB states [67,92,93]. Thus, the role of *L. iners* in VMB’s health and the association between *L. iners* dominant VMB (CST III) and genital pathogens during pregnancy should be further evaluated.

## 5. Limitations

The main limitation of this study was the limited availability of longitudinal paired samples. This was mainly due to practical challenges in the sample collection process. Currently, only vaginal samples from two-woman sampled at all three-collection timepoints were available for testing. Nevertheless, the findings of this first analysis in pregnant Pemban women raise many questions (regarding the role of VMB with pathobionts, genital infections and pregnancy outcomes) that could be further investigated in a bigger cohort. In this study, the number of participants was limited to 90 and, as such, the prevalence of adverse pregnancy outcomes was low. However, pregnancy complications are prevalent in Pemba, and the role of VMB in pregnancy outcomes should be further investigated in this population in a bigger cohort [94]. This study does nonetheless provide baseline characteristics, including clinically relevant ones, and insights on pregnancy outcomes among women in Pemba Island. Unfortunately, due to the use of pre-existing biobank data, some information, such as BV-related clinical data, that would have been relevant to our aim was not among the information available from the questionnaire.

With regards to the methodological approaches used in this study, the IS-pro assay gives comparable results to next-generation sequencing (NGS) and has been shown to even outperform NGS when using samples with lower bacterial loads [61,95]. 16S RNA gene sequencing is currently the most used method to the determine the microbiota of vaginal samples. Singer et al., compared vaginal samples of IS-pro and 16S RNA gene sequencing and showed that the vast majority of samples had a similar profile in terms of alpha diversity [96]. Not all bacterial taxa to species level have been identified due to restrictions of the available database. However, this mainly related to low-abundant organisms. For instance, *L. vaginalis* could not be identified yet in this analysis.

The use of antibiotics and twin pregnancies, both factors of which can interfere with VMB composition, were only reported by a small number of women and therefore were not excluded from this analysis.

Finally, the use of the term “pathobiont” in this study and other studies should be taken carefully, as it is not a well-defined concept [97]. It has been shown in some studies that the conditions under which pathobionts exhibit virulence relate to impaired host immune defences or altered microbiota compositions [97]. This in turn suggests an agreement on what a balanced VMB state or an alter VMB state are [55]. On the other hand, the clinical significance of VMB compositions remains debated, especially among women from sub-Saharan Africa or African ancestry [48,97,98].

## 6. Conclusions

The VMB was generally *Lactobacillus* dominant (65% at <20 weeks GA and 81% at the ≥20 weeks GA timepoint) during pregnancy and non-*Lactobacillus* dominant (73.9%) post-delivery in women from Pemba Island. The VMB richness significantly decreased during pregnancy in paired and unpaired samples, whereas the Shannon diversity significantly increased post-delivery solely in unpaired samples. A substantial presence of pathobionts was observed in a diverse (or *L. iners*-dominant) VMB at all three timepoints. Solely at the ≥20 weeks GA pregnancy collection, genital infection positively associated with *L. iners* dominant VMB. Future studies should further investigate the temporal and directional microbial changes during pregnancy, the role of CSTs with adverse pregnancy outcomes and how pathobionts and genital pathogens interfere with the VMB composition, especially with *L. iners* dominant VMB.

Investigating the causality of particular VMB characteristics in reproductive health might be essential to improve maternal and child health, particularly in populations where the burden of VMB dysbiosis, genital infections and adverse pregnancy outcomes are high, such as in certain parts of sub-Saharan Africa. Once clarity is provided on beneficial and non-beneficial VMB in pregnant women of different ethnicities, better and cheaper diagnostics tools and treatment options (probiotics or other biotherapeutics products) can be investigated for more personalized clinical solutions.

## Figures and Tables

**Table 1:** Description of the sociodemographic, clinical and pregnancy outcomes across cohort.

Characteristics	# of Total Women	Descriptive
Mother’s Age (years)	90	29.9 ± 6.6 (16–45) (Mean ± SD (range))
15–24		*n* = 21 (23.3%)
25–29		*n* = 27 (30%)
30–34		*n* = 16 (17.8%)
35–39		*n* = 16 (17.8%)
40–49		*n* = 10 (11.1%)
Gravidity	88	5.2 ± 2.6 (1–11) (Mean ± SD (range))
Parity	82	4.0 ± 2.4 (0–9) (Mean ± SD (range))
Number of first pregnancy	88	*n* = 6 (6.8%)
Maternal age at first pregnancy	80	19.8 ± 4.0 (15–32) (Mean ± SD (range))
Maternal body mass index	81	25.35 ± 5.29 (17.54–41.8) (Mean ± SD (range)) *n* = 6 (7.4%) Underweight (<18.5) *n* = 37 (45.6%) Normal weight (18.51–24.9) *n* = 23 (28.4%) Overweight (25.0–29.9) *n* = 15 (18.5%) Obese (>30)
Ethnicity	90	*n* = 90 (100%) Shirazi (Zanzibar Africans)
Religion	88	*n* = 88 (100%) Muslim, Islam
Number of school years completed	88	2.2 ± 1.2 (1–5) (Mean ± SD (range)) *n* = 23 (26.1%) one year *n* = 52 (59.1%) two years *n* = 1 (1.1%) three years *n* = 12 (13.6%) five years
Never Smoked	88	*n* = 88 (100%)
Diet	88	*n* = 1 (1.1%) followed diet pre-pregnancy *n* = 1 (1.1%) followed diet in the past 3 months *n* = 1 (1.1%) is currently following a diet *n* = 85 (96.6%) did not follow a diet
History with complicated pregnancy	79 *	*n* = 14 (17.7%) had one or more complicated pregnancy
		Separately: *n* = 9 (11.4%) had one stillbirth *n* = 1 (1.2%) had three stillbirths *n* = 2 (2.5%) experienced PROM *n* = 3 (3.8%) ever had a preterm delivery

PROM: premature rupture of membranes; SD: standard deviation. * *n* = 6 women were known to be on their first pregnancy and therefore excluded from calculations.

**Table 2:** Mean gestational age at each swab collection.

Collection Timepoint	# of Total Women	Gestational Age (Mean ± SD (Range))
<20 weeks GA pregnancy samples	44	118.2 ± 16.7 (69–139) days 16.9 ±1.3 (9.9–19.9) weeks
≥20 weeks GA pregnancy samples	82	193.5 ± 27.37 (168–280) days
Post-delivery samples	44	52.2 ± 7.9 (42–72) days

**Table 3:** Delivery information and pregnancy outcomes.

Delivery Information	# of Total Women	Descriptive
Outcome of delivery all included participants	90	*n* = 83 (92.2%) single live born *n* = 2 (2.2%) multiple births *n* = 4 (4.4%) miscarriage/abortion *n* = 1 (1.1%) Still birth
		*n* = 6 (6.7%) preterm delivery of which one are twins
Outcome of delivery women with vaginal samples tested at post-delivery	44	*n* = 38 (86.4%) single live born *n* = 2 (4.5%) multiple births *n* = 4 (9.1%) miscarriage/abortion *n* = 0 (0%) Still birth
The baby was ill after delivery (<48 h)	44	*n* = 1 (2.3%)

**Table 4:** Number of vaginal samples positive for urogenital pathogen within a community state type.

	CST I	CST II	CST III	CST IV	CST V	Total (%)
Samples of <20 weeks GA collection point						
CT	1/15	0/0	1/12	1/11	1/6	4/44 (9.1)
TV	1/15	0/0	1/12	0/11	0/6	2/44 (4.5)
HPV others	1/15	0/0	1/12	1/11	0/6	3/44 (6.8)
urogenital pathogens combined	3/15	0/0	3/12	2/11	1/6	9/44 (20.5)
Samples of ≥20 weeks GA pregnancy collection point						
CT	1/18	0/2	3/38	1/11	0/13	5/82 (6.1)
TV	1/18	1/2	5/38	0/11	1/13	8/82 (9.76)
MG	0/18	0/2	3/38	0/11	0/13	3/82 (3.7)
HPV others	1/18	0/2	2/38	0/11	1/13	4/82 (4.8)
urogenital pathogens combined	3/18	1/2	12/38 ^a,b^	1/11	2/13	19/82 (23.2)
Samples of post-delivery collection point						
CT	0/3	0/0	1/11	0/29	0/1	1/44 (2.3)
TV	0/3	0/0	1/11	2/29	0/1	3/44 (6.8)
NG	0/3	0/0	0/11	1/29	0/1	1/44 (2.3)
HPV others	0/3	0/0	1/11	3/29	0/1	4/44 (9.1)
urogenital pathogens combined	0/3	0/0	3/11	6/29	0/1	9/44 (20.5)

The number of positive samples/total samples tested. CST: community state type; CT: *C. trachomatis;* HPV: human papillomavirus (Genotypes 31, 33, 35, 39, 45, 51, 52, 53, 56, 58, 59, 66 and 68); MG: *M. genitalium;* TV: *T. vaginalis*. ^a^ The difference between the number of urogenital infections is significantly higher in CST III VMB compared with all the other CSTs (*p* = 0.01). ^b^ One person had a co-infection with HPV others and TV at CST III.

## Data Availability

Data are available from the authors with the permission of the Biobank governing body/local institution and the Principal Investigator of the site.

## Supplementary Materials

The following supporting information can be downloaded at: https://www.mdpi.com/article/10.3390/microorganisms10030509/s1. Figure S1: Number of vaginal samples at each collection point; Figure S2: Cluster analysis 44 vaginal samples collected during pregnancy at fist pregnancy collection; Figure S3: Cluster analysis 82 vaginal samples collected during pregnancy at ≥20 weeks GA pregnancy collection; Figure S4: Cluster analysis 44 vaginal samples collected post-delivery; Figure S5: The frequency of bacterial species or unknown genus/family bacteria observed per collection time point; Figure S6: Heatmap of all samples of the cohort in Pemba Island showing the 29 species with the highest relative abundance; Figure S7: Boxplot for the richness (A) and Shannon diversity index (B) at nucleotide level for each collection point; Figure S8: Boxplots for nucleotide richness at each collection point for paired samples; Figure S9: Boxplots for Shannon diversity index at nucleotide level for paired samples.
