# Supplementary material for: The Vaginal Microbiota Composition and Genital Infections during and after Pregnancy among Women in Pemba Island, Tanzania
Source: Microorganisms. 2022 Feb 25;10(3):509. doi: 10.3390/microorganisms10030509 (PMC8951098; doi:10.3390/microorganisms10030509)
Supplement: Supplementary file 1 [file microorganisms-10-00509-s001.zip › microorganisms-1537455-SI.pdf]

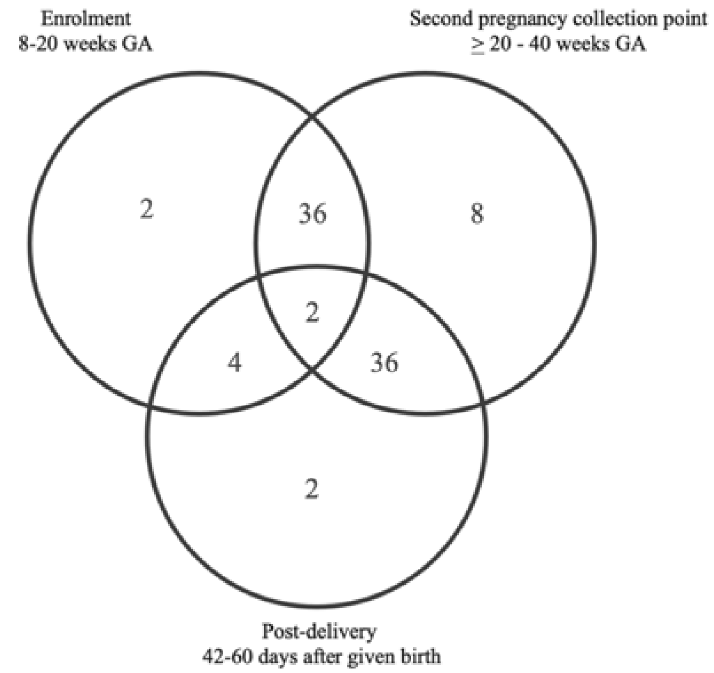

Figure S1. Number of vaginal samples at each collection point. In total 170 vaginal samples from 90 women were tested and analysed, 44 vaginal samples at < 20 weeks GA, 82 at ≥ 20 weeks GA pregnancy collection point and 44 post-delivery.

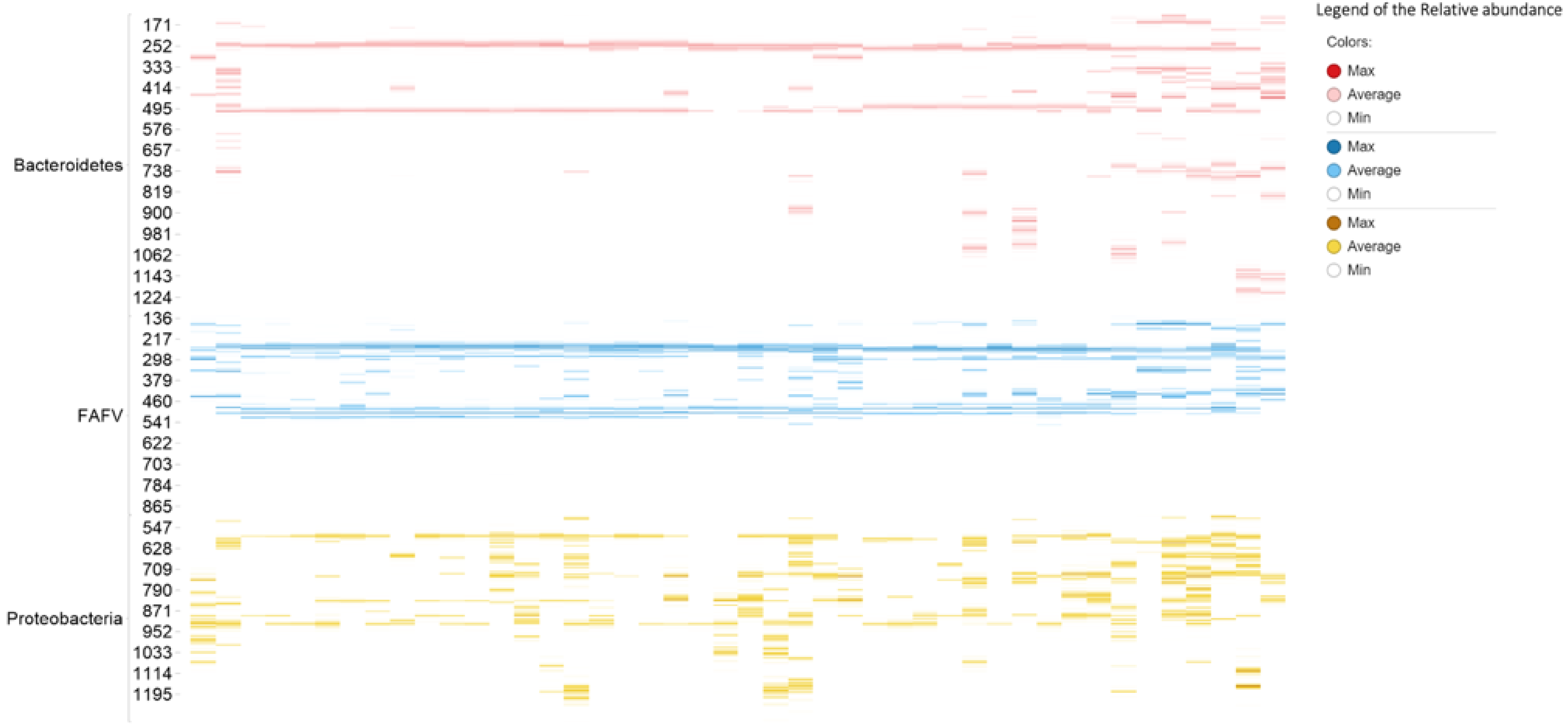

Figure S2. Cluster analysis 44 vaginal samples collected during pregnancy at fist pregnancy collection. Each column represents a sample; each row represents a bacterial species corresponding to a specific nucleotide number (bacteria from the phyla *Bacteriodetes* in red, *Actinobacteria, Fusobacteria, Firmicutes, and Verrucomicrobia* in blue, bacteria from the phylum *Proteobacteria* in yellow.

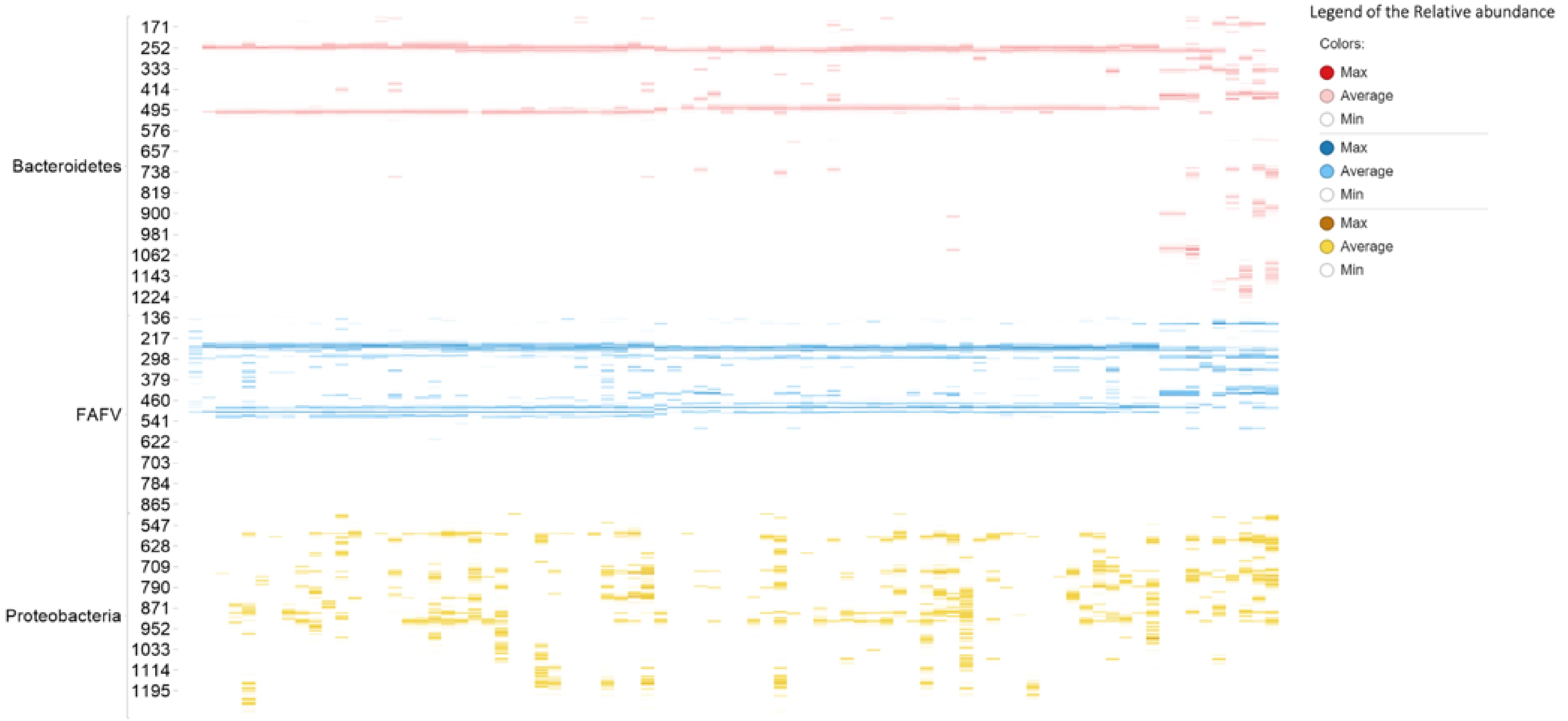

Figure S3. Cluster analysis 82 vaginal samples collected during pregnancy at ≥ 20 weeks GA pregnancy collection. Each column represents a sample; each row represents a bacterial species corresponding to a specific nucleotide number (bacteria from the phyla *Bacteriodetes* in red, *Actinobacteria, Fusobacteria, Firmicutes, and Verrucomicrobia* in blue, bacteria from the phylum *Proteobacteria* in yellow.

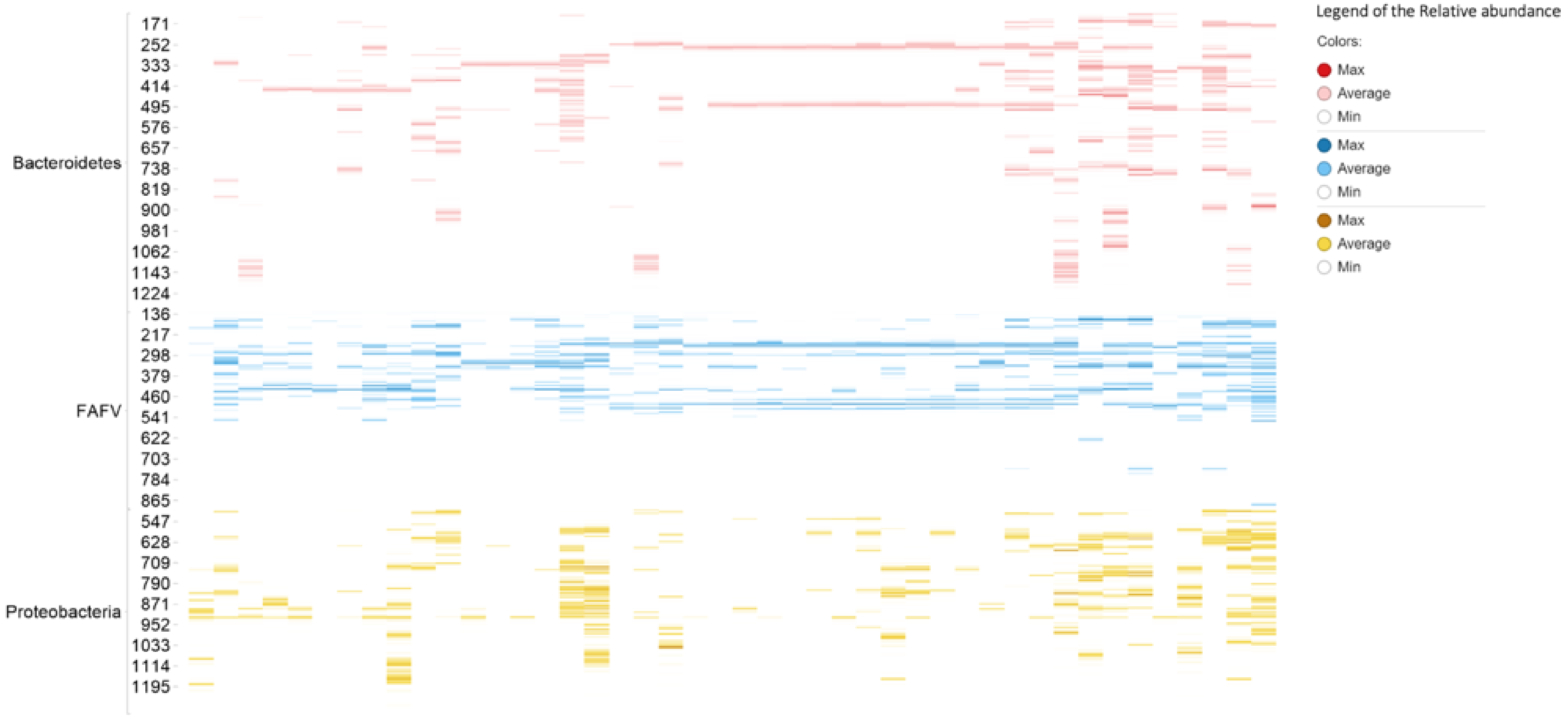

Figure S4. Cluster analysis 44 vaginal samples collected post-delivery. Each column represents a sample; each row represents a bacterial species corresponding to a specific nucleotide number (bacteria from the phyla *Bacteriodetes* in red, *Actinobacteria*, *Fusobacteria, Firmicutes, and Verrucomicrobia* in blue, bacteria from the phylum *Proteobacteria* in yellow.

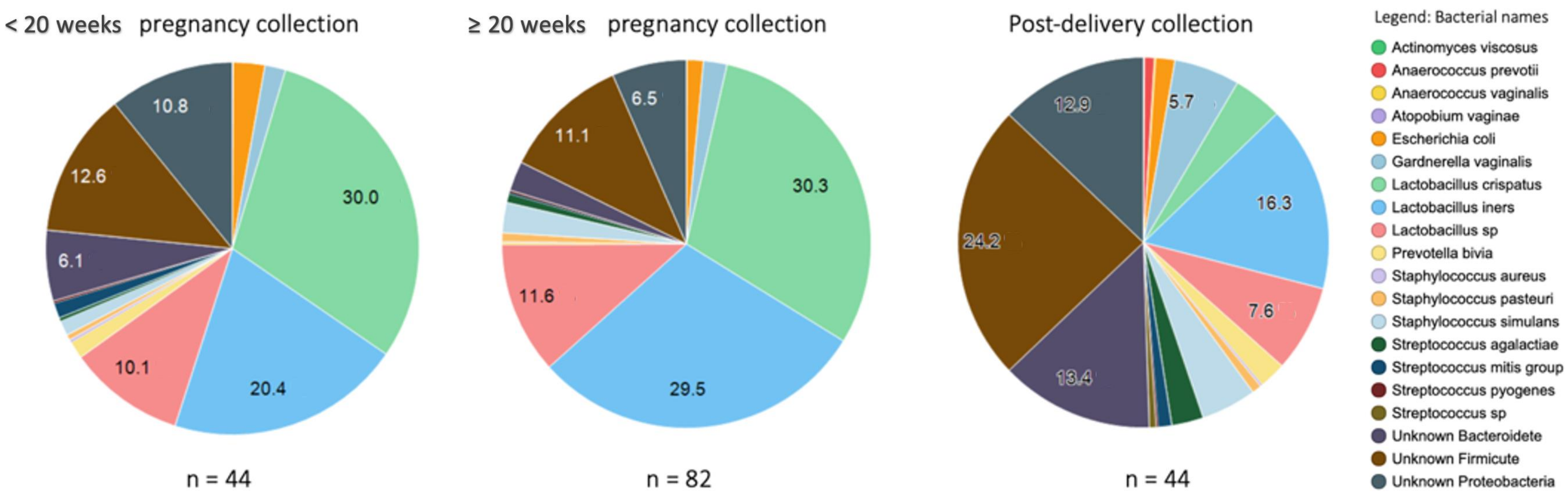

Figure S5. The frequency of bacterial species or unknown genus/family bacteria observed per collection time point.

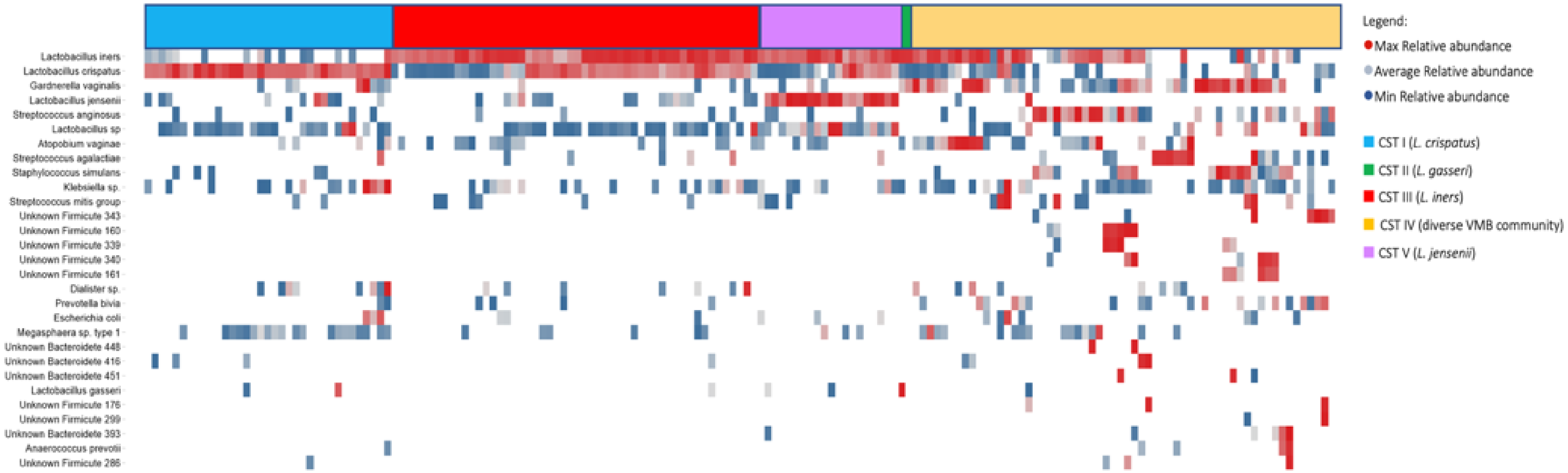

Figure S6. Heatmap of all samples of the cohort in Pemba Island showing the 29 species with the highest relative abundance. Each column represents a sample, each row represents a bacterial species In the dendrogram the community state type (CST) cluster are given in colour; CST I; blue, CST II; green, CST III; red, CST IV, yellow, CST V; purple

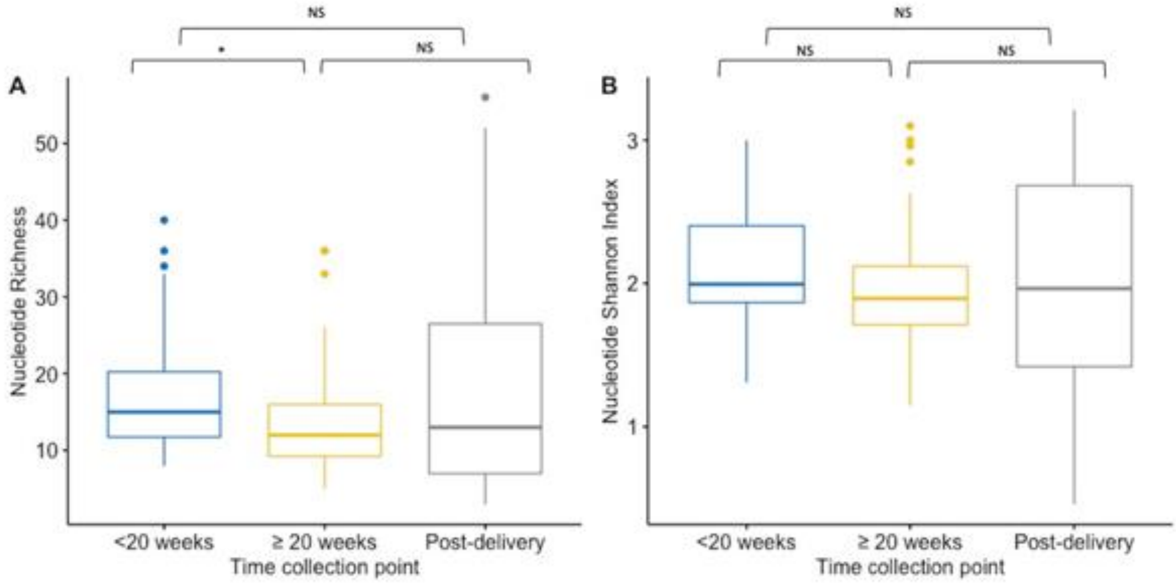

Figure S7. Boxplot for the richness (A) and Shannon diversity index (B) at nucleotide level for each collection point. Results of the < 20 weeks GA pregnancy collection are in blue (n = 44), ≥ 20 weeks GA pregnancy collection in yellow (n = 82), and post-delivery in grey (n = 44). A. Nucleotides richness is lower at ≥ 20 weeks GA pregnancy collection compare to the pregnancy collection at < 20 weeks GA pregnancy collection (p = 0.02), but between the other timepoints there were no significance difference. B. The Shannon diversity index did not significantly differ between the collection timepoints.

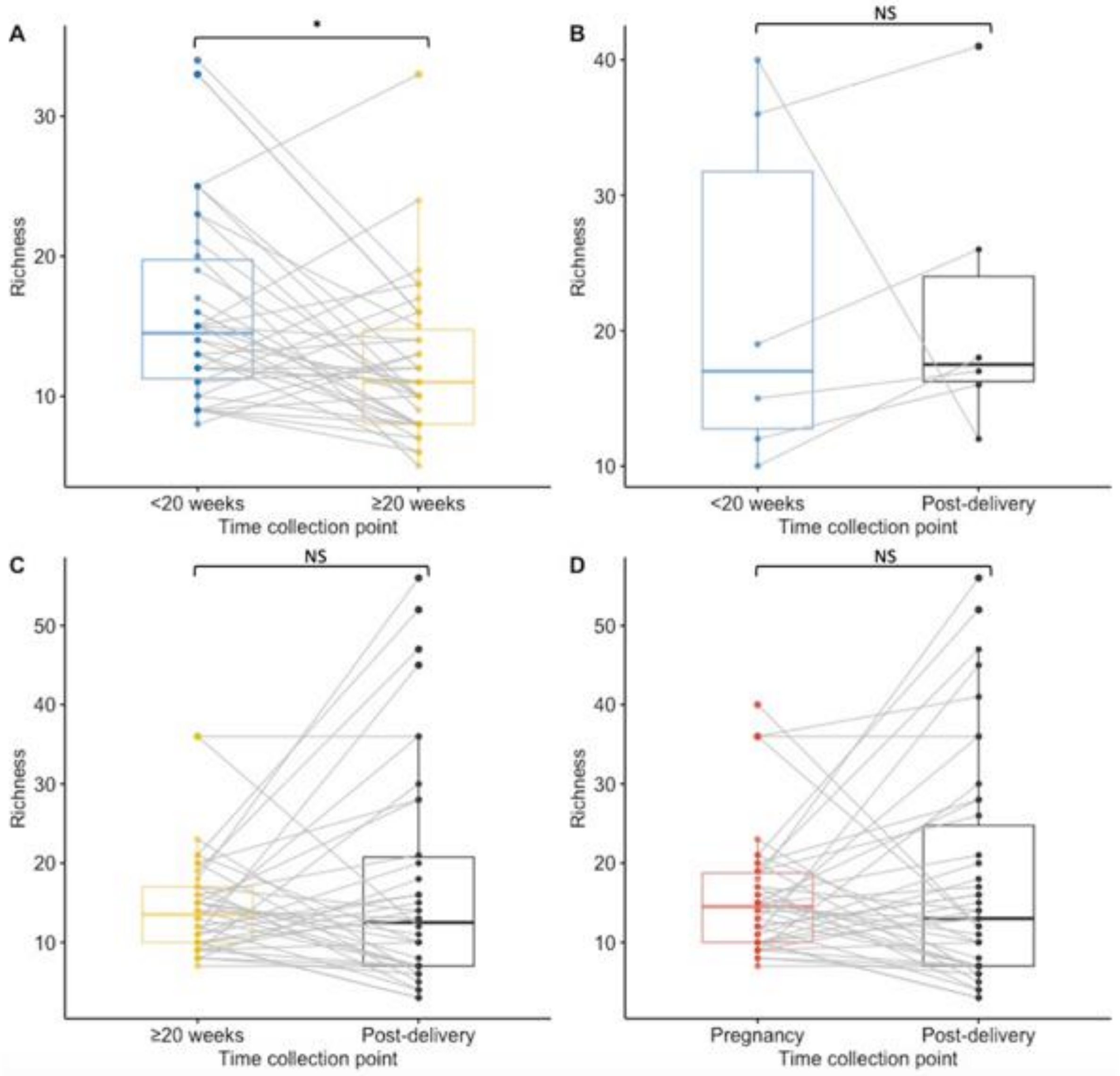

Figure S8. Boxplots for nucleotide richness at each collection point for paired samples. Results of the < 20 weeks GA pregnancy collection are in blue, ≥ 20 weeks GA pregnancy collection in yellow, and post-delivery in black and pregnancy in red. A. The nucleotide richness was significantly lower at the ≥ 20 weeks GA pregnancy collection point than at < 20 weeks GA pregnancy collection ($p < 0.01$) in matched samples from 38 women. B. There was no significant difference in the nucleotide richness between the < 20 weeks GA pregnancy collection and post-delivery matched samples from 6 women. C. No significant difference was calculated in the nucleotide richness between ≥ 20 weeks GA pregnancy collection and post-delivery matched samples from 38 women. D. For 42 women that had samples collected at least once during pregnancy and post-delivery, no significant difference in the species richness was calculated.

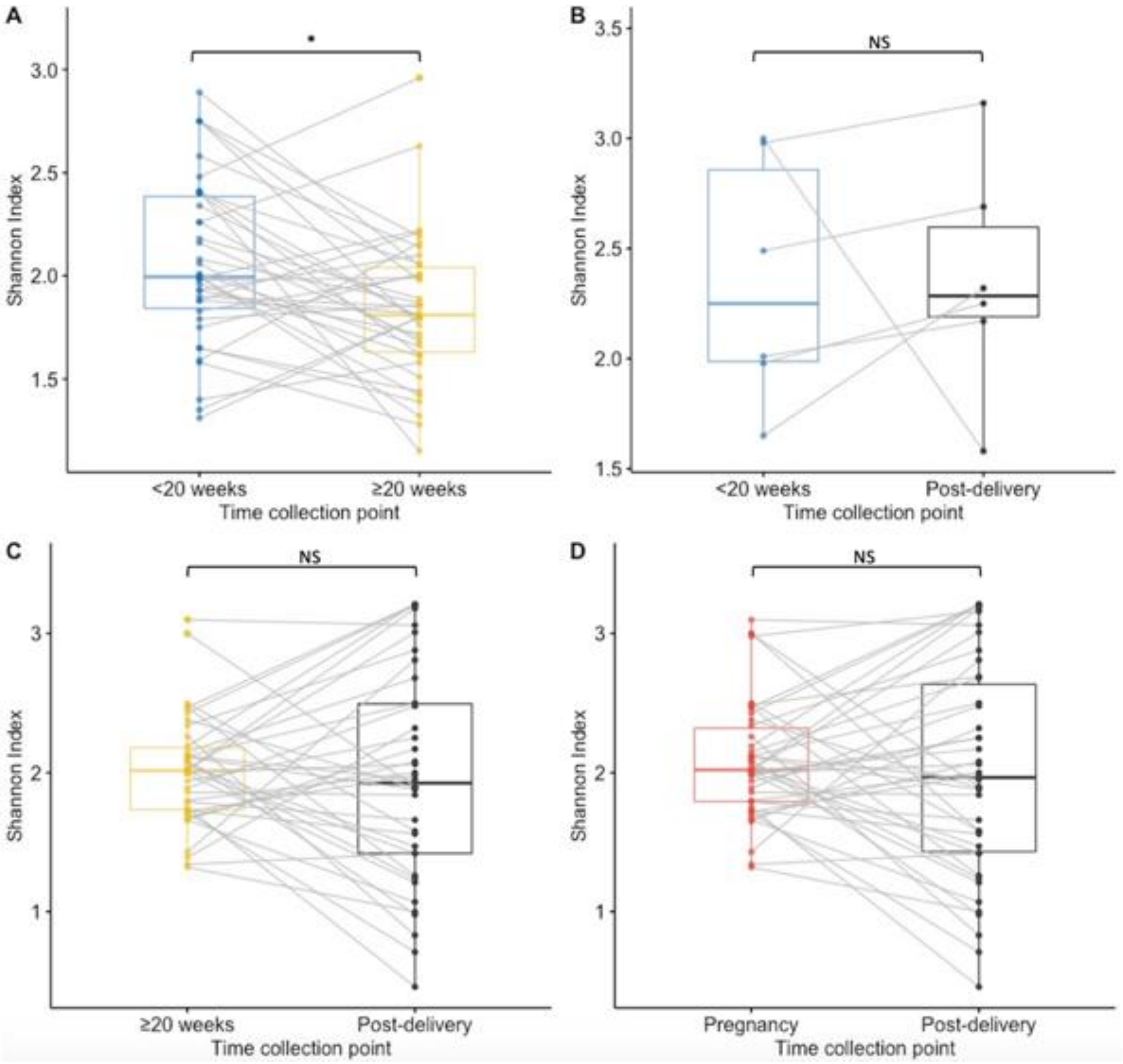

Figure S9. Boxplots for Shannon diversity index at nucleotide level for paired samples. Results of < 20 weeks GA pregnancy collection are in blue, ≥ 20 weeks GA pregnancy collection in yellow, and post-delivery in black and pregnancy in red. A. The Shannon diversity index was significantly higher at the ≥ 20 weeks GA pregnancy collection point than at the < 20 weeks GA pregnancy collection in matched samples from 38 women ($p < 0.01$). B. There was no significant difference in the Shannon diversity index between < 20 weeks GA pregnancy collection and post-delivery matched samples from 6 women. C. No significant difference was calculated in the Shannon diversity between ≥ 20 weeks GA pregnancy collection and post-delivery matched samples from 38 women. D. For 42 women that had samples collected at least once during pregnancy and post-delivery, no significant difference in the Shannon diversity index was calculated.
